# Risks of snakebite and challenges to seeking and providing treatment for agro-pastoral communities in Tanzania

**DOI:** 10.1371/journal.pone.0280836

**Published:** 2023-02-10

**Authors:** Monica Fredrick Francis, Sr. John-Mary Vianney, Kathrin Heitz-Tokpa, Katharina Kreppel

**Affiliations:** 1 Department of Global Health and Biomedical Sciences, School of Life Sciences and Bio-Engineering, Nelson Mandela - African Institution of Science and Technology, Arusha, Tanzania; 2 Centre Suisse de Recherches Scientifiques en Côte d’Ivoire, Abidjan, Côte d’Ivoire; 3 Department of Public Health, Institute of Tropical Medicine, Antwerp, Belgium; Universidad de Costa Rica, COSTA RICA

## Abstract

**Background:**

Continuous occurrence of snakebite incidences and the vulnerability of some communities remain a critical problem in sub-Saharan Africa. Despite causing permanent disability to almost half a million people annually and numerous deaths, snakebite and associated complications are still largely neglected. This study aimed at elucidating risk factors associated with snakebite cases, treatment availability and case management practices for vulnerable agro-pastoralist communities in Northern Tanzania.

**Methods:**

Data was collected in the Monduli (Arusha region) and the Simanjiro (Manyara region) districts in Tanzania. Interviews with 101 snakebite victims or their guardians and 13 health professionals from 3 health centers in the districts were conducted. Additionally, case records of patients admitted between 2007 and 2019 to the Meserani Snakebite Clinic were obtained.

**Results:**

This study showed that appropriate treatment for snakebite including anti-venom, is difficult to access and that snakebite incidences were significantly linked to factors such as gender, age, socio-economic activity, season of the year, and whether being at home or out in the fields. Anti-venom and trained health professionals were only available at the Meserani Snake Park Clinic. Men were bitten most often (χ^2^ = 62.08, df = 4, p-value < 0.0001). Overall, adults between the ages of 18 and 60 years (χ^2^ = 62.08, df = 4, p-value < 0.0001) received most bites, usually while outdoors herding cattle in the dry season. A significant majority of victims looked for traditional treatment first (52.7%, χ^2^ = 29.541, df = 2, p-value = 0.0001). The results of this study present crucial information on what is needed to improve the accessibility to appropriate treatment after a snakebite among agro-pastoral communities.

**Conclusion:**

The situation regarding morbidity and mortality due to the inaccessibility of common treatment for snakebite in northern Tanzania is challenging. Reliance on traditional medicine exacerbates the situation. There is dire need to involve affected communities, researchers, the government, clinicians and the public in general, to work together and take part in the global snakebite initiative. Communities and health professionals recognise the underlying challenges and have valuable suggestions on how to improve the situation.

## Introduction

Snakebite causes an estimated 81,410 to 137,880 deaths and about 413,000 permanent disabilities every year globally, and in 2017, it was recognized as a priority neglected tropical disease [1]. Snakebite is referred to as an injury caused by the *bite* of a *snake*, especially dangerous when from a venomous snake. These injuries are mainly affecting vulnerable and poor populations in rural settings of Africa, Asia and Latin America [2] Famous global venomous snakes include the black mamba, cobra, banded krait, saw-scaled viper and rattlesnake, and so far, 137 species of dangerous snakes have been reported [3]. Snake venom can be classified into three types, cytotoxins, neurotoxins, and hemotoxins. Each type results in different symptoms in humans and affects the body differently over time [4, 5].

In Africa alone, there are an estimated 435,000 to 580,000 snakebites annually [6–8]. Five venomous snake species are reported to have the highest medical impact in East Africa, including Tanzania; These include the spitting cobras (*Naja nigricollis* and *Naja mossambica*), the neurotoxic mambas (*Dendroaspis angusticeps* and *Dendroaspis polylepis*) and the puff adder (*Bitis arietans*) [4, 9].

Unfortunately, to date, snakebite and associated disease in African countries are still largely underestimated partly due to difficulties victims experience to reach formal healthcare services and also due to over-reliance on traditional treatments. While awareness of the problem is increasing among health professionals and public health experts, the situation is not adequately addressed. This is partly due to lack of baseline information on the challenges faced by victims and health care providers.

In Tanzania, snakebite is particularly a threat to rural communities where herding and agricultural activities are the major economic activities. Snake envenoming often causes serious morbidity and sometimes death. Snakebite may also have long-term psychological implications for victims, such as anxiety, depression, and post-traumatic stress disorder (PSTD) which in turn may result in social disadvantage and loss of employment due to disability [10]. This further emphasizes the mental, physical and socio-economic impact of snakebite for the victims and their communities.

The scale of snakebite envenoming and its impact on health is a function of several factors such as abundance of snakes, agricultural and pastoralist activities, limited availability and accessibility of antivenom (AV), and inadequately trained health workers for attending to this medical emergency [4]. Understanding the epidemiology of snakebite in the study area is important in order to fully appreciate the situation. In light of the challenges linked to access conventional treatment, victims in these communities have developed a long tradition of using herbal medicine and rituals as an alternative cure [2].

Despite observed health impacts to the communities, there are limited evidence-based studies on the management of snakebite and available medical care to victims in the country. Snakebite management would therefore benefit from a better understanding i) of the challenges to receive and provide treatment, ii) of the real burden and population at risk as well as iii) of the bite circumstances. This can provide essential information to advise public health experts to design strategies relevant to bite prevention and improved treatment.

Agro-pastoral and pastoral communities in the Arusha and Manyara regions in northern Tanzania are living daily with the risk of snakebite. This study, therefore, examined characteristics of bite victims, their health seeking behaviour and the perceived capacity of medical staff and traditional healers to treat snakebite, in order to identify treatment challenges. Undeniably, deadly snake species in Tanzania pose a silent health threat, which needs to be further explored to improve interventions in the interest of communities but also biodiversity.

## Materials and methods

### Ethics

Permission to carry out this study was granted by the district directors of the respective areas, village chairpersons of the visited villages, and the ethical review board of the consortium of the Kibong’oto Infectious Diseases Hospital, the Nelson Mandela African Institution of Science and Technology and the Centre for Educational Development in Health, Arusha (KIDH-NM-AIST-CEDHA)–(KNCHREC) with research permit number: KNCHREC 00046/03/202. All participants gave informed. written consent before taking part. For participants under 18 years of age, written consent was obtained from their parent/guardian.

### Study area and population

This map was created by the lead author. The map of Tanzania indicating regional borders is open access and was downloaded from vector maps (vemaps.com/tanzania/tz-02). Local borders were extracted using QGIS software, loaded onto the freely available software; Inkscape version 1.2.2, and the local points were marked on GIS extracts.

**Fig 1:**
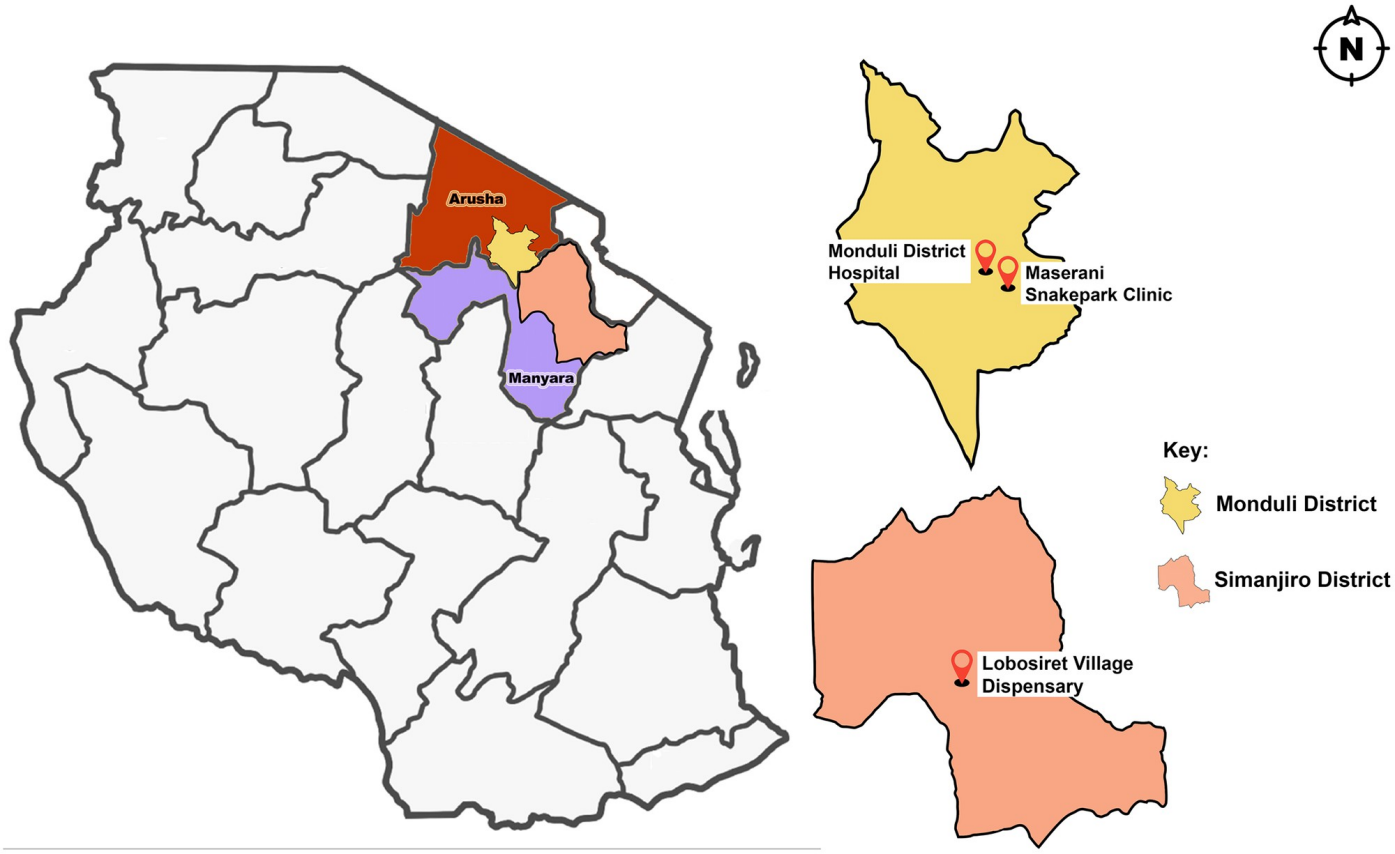
Map of the United Republic of Tanzania showing Monduli district (yellow) situated in the Arusha region (brown) and the Simanjiro district (pink), situated in the Manyara region (purple). Health facilities included in the study are marked on separate maps of Monduli and Simanjiro. (Created by Monica Fredrick Francis).

The Monduli district as shown in Fig 1 is one of six districts of the Arusha region, found in the northeastern zone of Tanzania, with an area covering 6,993km^2^. It is situated at 1,534 meters above sea level, at 3^◦^ to 4.50^◦^ south and 36.50^◦^ to 36.45^◦^ east. It is bordered to the north by Longido district, to the east by Arusha rural district, to the south by the Manyara region and to the west by Ngorongoro district and Karatu district. Monduli is generally sparsely populated with a total population of 158,929 (Tanzania 2012 National Census).

The major ethnic group inhabiting the Monduli district is the “Maasai” people (97%), and the majority are semi-nomadic pastoralists [11]. Families live in homesteads, “bomas”, of 20–70 people. The two main ethno-linguistic groups, the pastoralist Kisongo Maasai and the agriculturalist Arusha, speak the local language “Maa” and are culturally very similar. Other ethnic groups live mostly in town settlements, and in cultivated areas permitting intensive agriculture. Major crops include maize, beans, rice, wheat/barley, banana and coffee. Traditionally, women carry out farm and household chores and care for the children, while men and adolescents roam the fields with livestock during the day. The district’s arid and semi-arid land supports an economy based on livestock, particularly cattle, central to the pastoral economy. The cattle provide meat, milk and blood, and are assets that are used as community contributions, serve in rituals, confer status and cement relationships.

The Simanjiro district as shown in Fig 1 is one of six districts of the Manyara region in Tanzania. It is bordered to the north by the Arusha region, to the north east by the Kilimanjaro region, to the south east by the Tanga region, to the south by the Kiteto district, to the south west by the Dodoma region and to the west by the Babati rural district. It is found 1,388 meters above sea level at 3° 52’ south and 36° 36’ east. According to the 2012 Tanzania National Census, the population of Simanjiro district was 178,693. Ethnic composition, language, culture and livelihoods are the same as in Monduli.

The Monduli district hospital is a government run hospital with 119 beds and 99 staff including 11 medical doctors, 11 nurses, and 35 other supporting staff. The hospital serves predominantly the communities of Monduli receiving 60 to 70 patients per day on average, but patients from neighboring districts are also accepted.

Lobosiret village dispensary is located in the Simanjiro district in the Manyara region. This dispensary is staffed by 1 medical doctor and 1 nurse, and provides routine checkups and treatment of minor injuries predominantly to the people of Lobosiret village.

The Meserani Snakebite Clinic is located in the Monduli district close to the main road leading to Arusha town. The clinic is linked to the Snake Park tourist attraction and offers free medical care for burn and snake bite victims. The clinic has 6 beds and is manned all year round by 2 nurses and a nursing assistant. The clinic is the only place where AV is readily available in northern Tanzania. The two nurses are from the local Maasai community. All proceeds from the Maasai Cultural Museum connected to the snake park go towards the clinic. Overlanders and supporters of the clinic regularly donate supplies when passing through.

The data collection process for this study took place for 10 days (May 2021 –June 2021) whereby 6 days were spent in the Monduli district in the Arusha region and 4 days in the Simanjiro district in the Manyara region. Data were collected by our study team (PI and assistant) visiting communities in the districts and interviewing staff from the Meserani snake park clinic, the district hospital in Monduli, and the village dispensary in Lobosiret in Simajiro district.

### Study design

This study collected both qualitative and quantitative data using purposive sampling. For the quantitative component, two sources of data were used. First, routine data collected by the Meserani Snake Park Clinic on their patients was accessed. This database was used to identify areas with high snakebite incidences and the most common snake species that reportedly affect the communities. Second, snakebite victims or their guardians or witnesses of snakebite were visited in their community and health professionals at the hospital/dispensary. A questionnaire collected data on socio-demographic and socio-economic characteristics of contacted bite victims or their guardian (see SM 5 (*questionnaire for victims or their guardians*) in S1 File). Collected data from the victims included information on snake bite incidences such as location, time of day, season, affected body part and recovery as well as perceived challenges of receiving treatment. Quantitative data from health professionals included data regarding their profession, training and usual handling of snake bite cases. Additional qualitative data were collected through semi-structured individual interviews (see SM 5 (*questionnaire for victims or their guardians*) in S1 File). Interview guides were developed so that interviews followed the same theoretical framework, while still allowing investigation of different facets of the research question. Interviews were done with victims or their guardian and health professionals. The objective of the qualitative interviews was to discuss particular aspects in depth, including the challenges faced to access or provide treatment.

### Data collection in the communities

Via purposive sampling, information of the village of origin of snakebite victims treated at the Meserani Snake Park Clinic was collected, and village elders from these villages asked to facilitate meetings with those victims or their guardians. Using the snowball sampling effect, additional snakebite victims from the same communities were identified. The purpose of the study was explained first to village leaders and then to victims or their guardians. Participation was on a voluntary basis following informed, written consent for the interview. Respondents were free to withdraw from the interview at any point.

Interviews were done in person at the victim’s village in a central location such as under a tree by the project’s Principal Investigator (PI) and a research assistant from the local community, who also administered and filled out the semi-structured questionnaire. During the interview, notes were taken by the research assistant and audio recorded. The local “Maasai” language or Swahili was used to ask questions on the demographics and Knowledge Attitudes and Practices (KAP) on snakebite as well as on the actions undertaken in case of snakebite and the experience of the health care received. Information was recorded in Swahili using an open data kit and later transcribed and translated into English. In total, 101 respondents living in two communities (37 in Monduli District and 64 in Simanjiro District) were interviewed to gather information about snake encounters, knowledge, attitude, management, and treatment practices of snakebite in the study area. For the questionnaires the team waited until it was completed. Interviews were held from 30 minutes to 1hour with those victims or guardians who agreed to be interviewed.

### Data collection in the health centers

The three health centers serving the communities in the Simanjiro and Monduli districts, the independently run Meserani Snake Park clinic and the Monduli district hospital, as well as Loibosiret village dispensary, were visited. From the Meserani Snake Park Clinic, case records of patients admitted between 2007 and 2019 to the clinic were obtained. The same project PI and research assistant that collected data from the victims administered a semi-structured questionnaire (see SM 6 (*questionnaire for health professionals*) in S1 File) in Swahili to health professionals and recorded the detailed responses from the semi-structured interviews on audio. For the questionnaires the team waited until it was completed. Interviews were held from 30 minutes to 1hour. Thirteen health professionals were interviewed in 3 health centers; 3 medical staff at Meserani Snake Park Clinic, 9 from Monduli District hospital and 1 nurse at Loibosiret village dispensary in Simanjiro to gather information about knowledge, treatment and management practices of snakebite cases. Participation was on a voluntary basis following informed written consent for the interview. Respondents were free to withdraw from the interview at any point. Questionnaires were filled out and interviews were held in quiet places away from patients and colleagues.

### Data analysis

All information from the questionnaires, patient records and interviews were translated into English by the Principal Investigator (PI) and entered into Microsoft Excel and Word. Data quality check was done by the PI’s supervisor. Quantitative analysis was carried out using R software version 3.6.1 (Core-Team. R: A language and environment for statistical computing. R Foundation for Statistical Computing, Vienna, Austria, 2019). Qualitative data were entered into QSR NVIVO version 12.5.0 (NVivo qualitative data analysis software; QSR International Pty Ltd. Version 12, 2018).

### Quantitative analysis

Descriptive analysis on the demographic characteristics of the snakebite victims interviewed in their communities was carried out, as well as on the information collected from the medical staff. Characteristics of the snakebite victims seen by nurses at the Meserani snake park clinic from 2007 to 2019 were summarized. Significant differences between groups were established using chi-square tests. The groups were gender, age-group, season, location of bite incident, activity and district.

### Qualitative analysis

The semi-structured interview data was transcribed and translated into English by the PI. The qualitative information was used to shed light on the challenges faced by individuals when seeking or providing health care for snakebite. For the qualitative data, a content analysis was conducted. In an iterative inductive process, the transcribed interviews were coded, and themes identified in relation to the research questions [12]. Some quotations were selected to illustrate aspects of the quantitative results. The particular focus was on challenges to seek and provide treatment for snakebites.

## Results

### Meserani Snake Park Clinic data

The database obtained from the Meserani Snake Park Clinic of recorded cases from the year 2007 to 2019, shows an increase in the number of patients from 5 to 76 per year, with more adults than children receiving treatment and more males than females (Table 1).

**Table 1:** The recorded number of snakebite cases presenting at Meserani Snakebite Clinic by gender and age from 2007 to 2019.

Year	Adults	Children	Males	Females	Total Number of Victims
2007	5	0	3	2	**5**
2008	21	15	23	13	**36**
2009	24	25	29	20	**49**
2010	33	16	31	18	**49**
2011	29	19	24	24	**48**
2012	36	21	29	28	**57**
2013	30	20	27	23	**50**
2014	42	31	32	41	**73**
2015	42	35	36	41	**77**
2016	34	32	37	29	**66**
2017	20	25	21	24	**45**
2018	32	30	36	26	**62**
2019	43	33	43	33	**76**
**TOTAL**	**391**	**302**	**371**	**322**	**693**

### Data from the communities

#### Characteristics of snake bite victims

**Fig 2:**
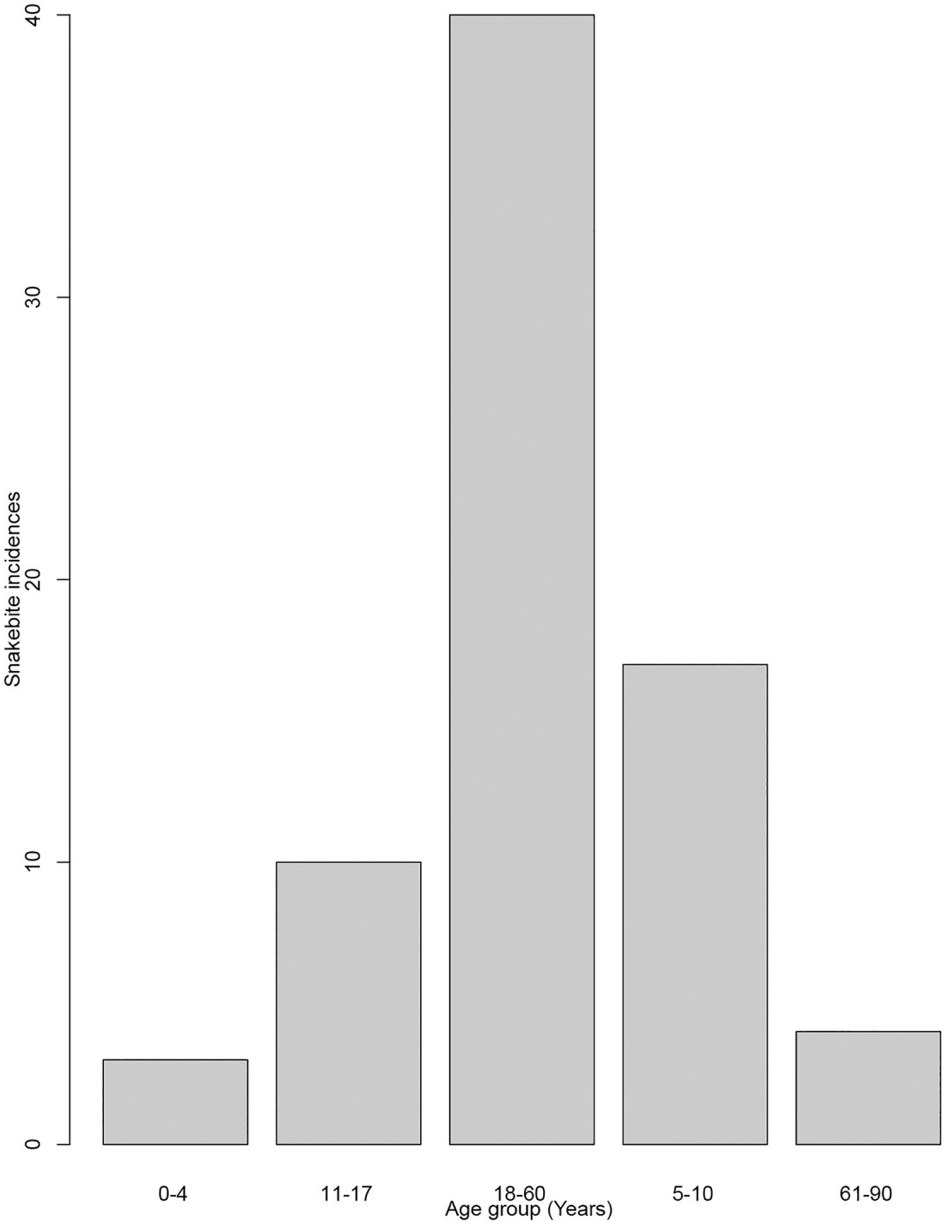
Snakebite incidences by age group.

**Fig 3:**
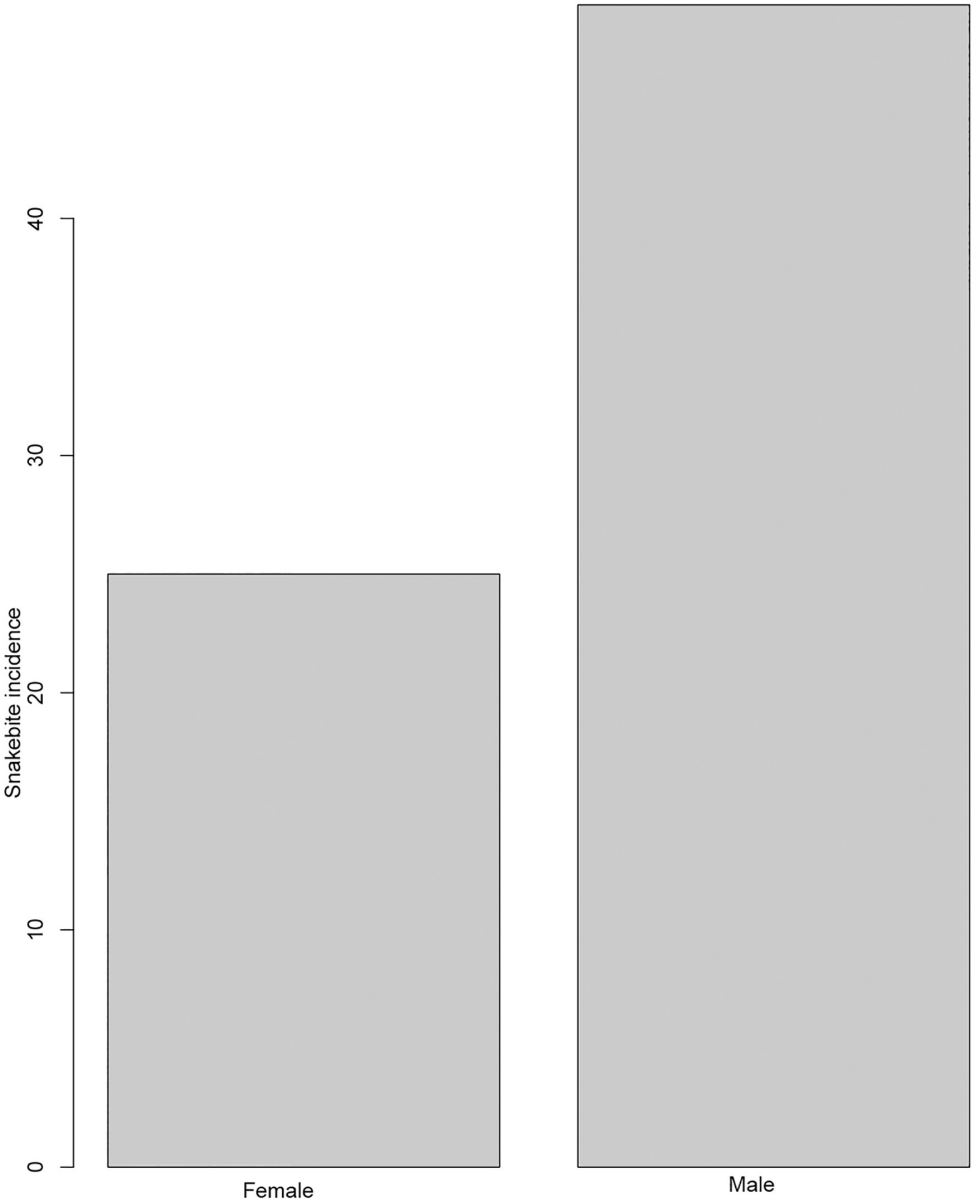
A graph showing the distribution of snakebite incidences by gender that were interviewed during the study from both districts.

A total of 101 respondents consisting of victims (n = 74), guardians or relatives of victims (n = 27) were interviewed. They either were victims of snakebite themselves or witnessed a snakebite and took care of the victim (Table 2). In total, information about 74 snakebite victims was collected, ranging from 8 months to 90 years old (mean age = 18 years; Table 2, Fig 2 (SM 4 in S1 File)). There were more male (66.2%) snakebite victims than female (33.8%). with. More males and females were bitten outside than inside their home (47.3% and 21.6% respectively, Fig 3 (SM 3 in S1 File)). The body parts most often affected were limbs (leg 55.4%, hand 23% and foot 14.9%, Table 3). Other body parts were affected to a much lesser extent. Activities carried out by the victims at the time of the bite included walking (36.5%), herding (27%), sleeping (16.2%), playing (8.1%), farming (6.8%), construction activities (4.1%), and sitting (1.4%) (Table 3). The reported health outcomes ranged from “completely healed” to “death” (Table 3). In seven cases the victim had died following the bite. Overall, ninety percent of victims reported symptoms after the bite, such as trauma associated with severe pain at the bite site, bleeding, body swelling, skin discoloration, dizziness, vomiting, sweating, high fever, tremor, or unconsciousness. Furthermore, two (3%) of the victims were left with a permanent disability, two (3%) have scars, while thirty-three (45%) still experience pain at the affected area (Table 3).

**Table 2:** Socio-demographic characteristics of respondents (n = 101) and snakebite victims the respondents referred to (n = 74) from the Monduli and the Simanjiro districts in northern Tanzania (May 2021-June 2021).

	*Respondents (n = 101) (74 bite victims + 27 guardians/witnesses)*	*Bite victims (n = 74)*
***Sex***	***n (%)***	***n (%)***
*Male*	*71 (70*.*3)*	*49 (66*.*2)*
*Female*	*30 (29*.*7)*	*25 (33*.*8)*
***Circumstance***		
*Personally bitten*	*74 (73*.*3)*	*74 (100)*
*Witness to bite*	*27 (26*.*7)*	*NA*
***Age group (years)***		
*0 to 4*	*0*	*3 (4*.*1)*
*5 to 10*	*0*	*17 (23)*
*11 to 17*	*0*	*10 (13*.*5)*
*18 to 60*	*22(21*.*8)*	*40 (54)*
*61 to 90*	*5(4*.*9)*	*4 (5)*

**Table 3:** Epidemiological characteristics of snakebite in the Monduli and Simanjiro districts in northern Tanzania and associated risk factors (n = 74 victims).

Characteristics	Frequency n (%)
**Activity**	
Construction	3 (4.1)
Farming	5 (6.8)
Grazing	20 (27.0)
Playing	6 (8.1)
Sitting	1 (1.3)
Sleeping	12 (16.2)
Walking	27 (36.5)
**Body part**	
Head	1 (1.3)
Cheek	1 (1.3)
Nose	2 (2.7)
Mouth	1 (1.4)
Hand	17 (22.9)
Leg	41 (55.4)
Foot	11 (14.9)
**Long-term effects**	
Death	7 (9.5)
Disability	2 (2.7)
Healed	30 (40.5)
Pain	33 (44.6)
Scar	2 (2.7)
**Time of day**	
Morning (6am–11 am)	4 (5.4)
Afternoon (12pm–4 pm)	30 (40.5)
Evening (5pm–6 pm)	11 (14.9)
Night (7pm–5 am)	29 (39.2)
**Initial treatment**	
Traditional	39 (52.7)
Hospital	32 (43.2)
None	3 (4.05)
**Season**	
Dry	71 (95.9)
Rainy	3 (4.1)
**Location**	
Home	23 (31.1)
Field	51 (68.92%)

**Fig 4:**
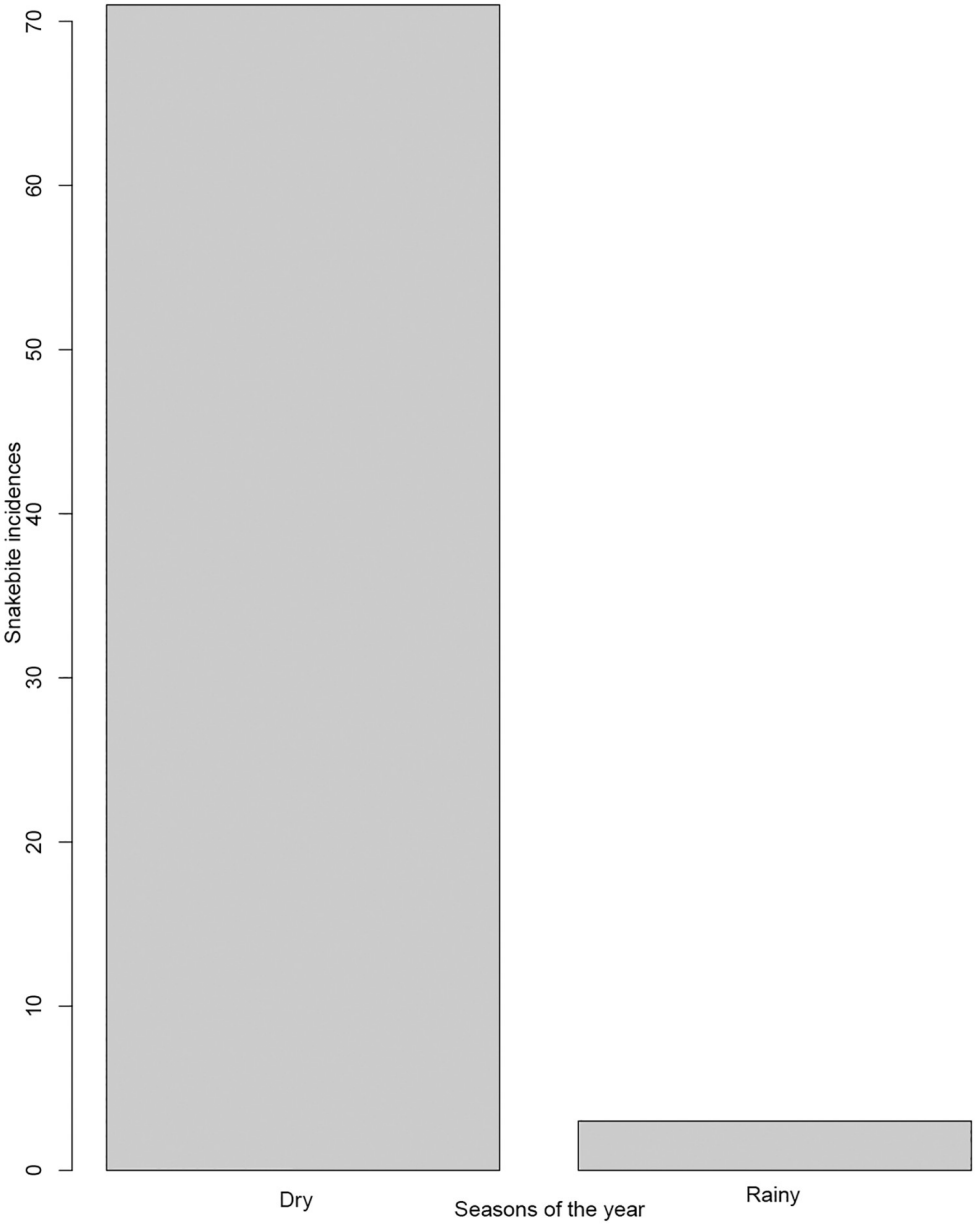
Distribution of snakebite incidences reported from the studied districts separated by season, with more incidences occurring more during the dry season.

Of the seventy-four snakebite victims, significantly more males (66.2%) than females (33.8%) were bitten (χ^2^ = 7.78, df = 1, p = 0.005; Table 4). Overall, significantly more victims, 68.9% (51/74), were bitten whilst out in the fields than while in their home stead (χ^2^ = 10.6, df = 1, p-value <0.0011; Table 4). Of those, a significant majority (54.06%; 40/74) were adults between 18 and 60 years old (χ^2^ = 62.08, df = 4, p-value < 0.0001, Table 4). Significantly more people were seeking traditional treatment (52.7%) than hospital treatment (χ^2^ = 29.54, df = 2, p-value <0.0001; Table 4). Significantly more bites (96%; Table 4) happened in the dry season (χ^2^ = 62.48, df = 1, p-value < 0.0001; Table 4, Fig 4 (SM 2 in S1 File)). There was a significant difference between activities (χ^2^ = 53.14, df = 6, p-value < 0.0001; Table 4), with people more likely to suffer snakebites when walking, than when doing any other activity.

**Table 4:** Chi square (χ^2^) values within each variable group of snake bite incidences.

Variable	Chi square value (χ^2^)	Degrees of freedom (df)	p-value
Age group (5 groups)	62.081	4	1.059e-12*
Gender (male or female)	7.7838	1	0.005272*
Season (wet or dry)	62.486	1	2.683e-15*
Activity (7 activities)	53.135	6	1.102e-09*
Location (inside or outside home)	10.595	1	0.001134*
Snakebite treatment (traditional or hospital)	29.541	2	3.849e-07*
District (Monduli or Simanjiro)	0.48649	1	0.4855

*Indicates variables showing a significant difference among the compared groups within the variables at p-value < 0.05.

**Fig 5:**
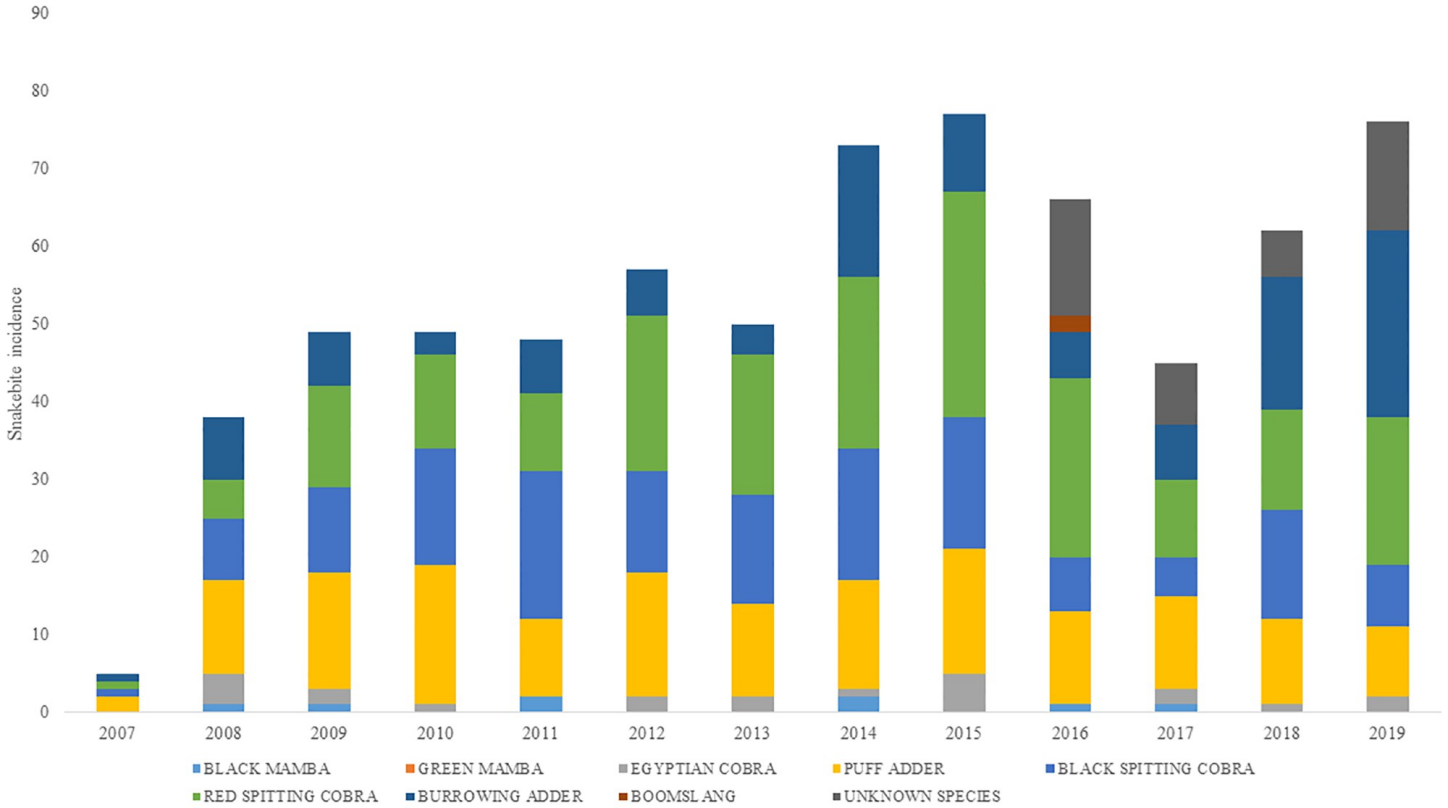
Snakebites by snake species recorded from snakebite victims admitted at Meserani Snake Park Clinic from 2007 to 2019.

Out of a hundred and one interviewees who participated in this study, twenty-eight reported that they could differentiate poisonous snakes form non-poisonous snakes. This was based on the snake’s appearance, specifically focusing on its skin color. Only 36 interviewees out of 101 had received any kind of training on snakes and how to offer first aid treatment to snake bite victims. These trainings were either part of school education, given by the Meserani Snake Park, or from the elders of the communities using traditional medicine. The snake species that were identified to have caused the bites seen at the clinic were Puff adders, Burrowing adder, Red spitting cobra, Black spitting cobra, Boom slang, Black mamba, Egyptian cobra and Green mamba (Fig 5 (SM 1 in S1 File)).

#### Characteristics of health centre employees

Thirteen health care workers (8 females, 5 males) were interviewed. This included nurses, nursing assistants, general practitioners, laboratory technicians and an anesthetist (Table 5). All but one had tertiary education, with a higher educational level seen at Monduli district hospital than the dispensary and the Meserani Snakebite Clinic. Overall, of those interviewed, only four (30.8%) had received any kind of training in the treatment of snakebite. Notably, at Monduli hospital, while overall more medical expertise is present, knowledge on snakebite treatment is low (1 out of 6 medical staff; Table 5). At the Meserani Snakebite Clinic two out of the three health care workers also trained the community on snakebite prevention and treatment, while in Monduli only one nurse did so. At Monduli hospital and Loibosiret dispensary only first aid was provided in case of snakebite and patients were sometimes referred to the Meserani Snake Park Clinic, where AV was available (Table 5).

**Table 5:** Demographic characteristics of the 13 health professionals interviewed at the Meserani Snake Park Clinic, the Monduli district hospital and the Loibosiret dispensary.

	Meserani Clinic	Monduli hospital	Loibosiret Dispensary (Simanjiro)
**Gender**			
Female	2	5	1
Male	1	4	0
**Age**			
20–40	1	9	1
41–60	1	3	0
**Education level**			
Primary	0	0	0
Secondary	1	0	1
Tertiary	2	9	0
**Occupation**			
General practitioner	0	1	0
Anesthetists	0	2	0
Nurse	2	5	1
Health care assistant	1	0	0
Lab technician	0	1	0
Pharmacist	0	1	0
**Knowledge on snakebite treatment**			
Yes	3	1	0
No	0	8	1
**Training the community on snakebite prevention and treatment**			
Yes	2	1	0
No	1	8	1
**Treatment for snakebite**			
Anti-venom availability	Yes	No	No
First aid only	No	Yes	Yes

### Qualitative results

#### Meserani Snake Park Clinic

*Research interest in snakebite*. Health care professionals at the Meserani Snake Park Clinic revealed that research interest in snakebite is relatively low and since its opening, only a small number of local researchers approached the clinic and only one institution of higher education from the United Kingdom.

*Treatment procedure*. When a victim reaches the Meserani Snakebite Clinic, the medical staff takes the history of the bite either from the patient or from accompanying relatives. The bite wound is inspected and the symptoms are observed to help with the identification of the species of snake that bit the victim, so that the appropriate treatment can be administered to the victim. This would be followed by wound washing and provision of AV for victims bitten by a venomous snake species. To manage bite symptoms as well as side effects of the administered AV, other drugs are given (including adrenaline, antihistamines and steroids).

*Treatment challenges*. Due to lack of medical health care services for snakebite close to the villages and due to limited availability and considerable costs of transport to the Meserani Snake Park Clinic, victims opt for traditional treatment by traditional healers close by. This was the case for 39 (52.7%) of the interviewed victims, while only 32 (43.4%) of the interviewed population could afford seeking hospital treatment.

Medical staff at the Meserani Snake Park Clinic reported difficulties deciding which AV to administer when not enough information is available to determine the snake species responsible for the bite. This occurs when a bite victim comes in unconscious, is a small child or has poor knowledge of snakes. Failure of victims to identify or describe the snake species responsible for the bite means staff has to rely on identifying the snake species solely by looking at the bite wound and the symptoms shown.

“Sometimes patients arrive at the clinic unconscious or they are children and they cannot tell us what species of snake it was”

(Nurse, Meserani Snake Park Clinic, In Depth Interview (IDI))

Even after identifying the species, administering treatment can be challenging due to allergic reactions that some of the patients develop as this quote confirms: “another problem are the allergic reactions to the AV. Treating these requires experience and the right drugs” (Nurse, Meserani Snake Park Clinic, IDI)

Another challenge for the medical staff is the availability of AV. The AV needs to be imported, usually from South Africa, what takes a long time. Especially since the COVID-19 pandemic started, it has taken over six (6) months to import AV from South Africa to Tanzania. Additionally, the clinic’s resources are strained and the clinic struggles to provide treatment for all patients, some of whom are referrals from other hospitals in northern Tanzania where training and medication to treat snakebite victims is lacking.

Other treatment challenges include the arrival of victims that did not come immediately after being bitten, either because they live far away or because they used traditional methods first. In these cases, the nurses have to deal with advanced stages of the snake bite effects such as systemic complications.

*Training the community*. The nurses at the clinic defined training on snakebite of individuals and/or the community as “teaching prevention and treatment measures to be given as a first aid to the victims”.

“We train those who come to visit the snake park on what to do once they are bitten by a snake, and also advise on prevention measures that they can follow to avoid snakebites in the future such as wearing closed shoes when they are out in the fields. Also, we emphasize to avoid sleeping in bushy areas where snakes are common, avoid walking in dark places and if possible using solar torches during the night”

(Nurse, Meserani Snake Park Clinic, IDI).

#### Monduli district hospital

*Attitudes of health professionals at the Monduli district hospital*. The medical staff interviewed during the survey, recommended improvements in the training of medical personnel and in the education of medical students through refresher courses on how to handle snakebite victims. This would enable correct administration of available AV required by the victims. The medical staff also suggested educating the community to enable them to administer first aid and equipping the village health centres with first aid kits for easy and immediate access whenever needed. Standard protocols and tools for diagnosis and treatment of snakebite were suggested by the clinicians. Implementation of integrated programs ensuring maintenance of proper storage facilities, appropriate training of health staff, and provision of AV and other drugs (including adrenaline, antihistamines and steroids) required for the management of envenoming. Preparation of national and regional snakebite treatment and management guidelines was suggested, in order to ensure suitable care for the victims.

Snakebite victims that arrived at the Monduli District Hospital, or the village dispensaries in these areas, received no treatment due to lack of AV. Instead, they were advised to go to the Meserani Snake Park Clinic for treatment where the AV is freely available.

“In the last 5 years, only six (6) victims have reported here for snakebite treatment but they were sent to the Meserani Snake Park Clinic due to lack of AV here at the Monduli District hospital”

(General practitioner, Monduli District hospital, IDI)

*Challenges for the village health center*. The health center in Loibosiret lacks appropriate treatment for snakebites including AV. This was attributed to the unaffordable costs of AV and treatments. Village health centers in northern Tanzania also lack proper AV storage facilities. This results in both village dispensaries and government referral hospitals in these districts lacking AV, thus victims are only given first aid and are then referred to the Meserani Snake Park Clinic. The majority of medical staff we interviewed also lacked knowledge and training on how to manage and treat a snakebite (69%).

#### Snakebite victims and guardians

*Difficulties to receive treatment*. Due to transport problems and cost, victims in these communities fail to get proper treatment on time once bitten. Victims and their guardians are required to travel long distances from their villages to the Meserani Snake Park Clinic, the only place where AV and expertise is currently available. The interviewed victims and guardians suggested that increased knowledge among the community members on first aid for snakebites from non-venomous and venomous snakes would substantially reduce suffering and fatal outcomes.

*Choosing traditional over hospital treatment*. The preferred transport from the remote villages in the study area is by motorbike due to bad roads and the long distance to the main road. When an incidence happens at night, the journey takes even longer than during the day, due to the unavailability of transport. The only other option then is to carry the victim for hours, what is only possible if it is a child. Traditional remedies used for snakebites however, are readily available such as the “black stone” carried by many Maasai herders and *40 KAMILI*, a traditional medicine found in powder form.

*Practices*. Due to lack of medical health care services for snakebite close to the villages, transport problems and costs, 52.7% (39/74) of victims opted for traditional healers and traditional treatment, while only 43.4% (32/74) of the interviewed population could afford seeking hospital treatment (Table 3). Thirty-nine (53%) victims received first aid immediately after the bite. When first aid was provided, the most common treatment was a piece of cloth used as a tourniquet together with the *snake stone*, made from a burnt piece of cow bone, applied after an incision at the wound is made. In other cases, the site of the bite was washed with cow’s milk or urine. First aid was given through wound incision at the site of the bite followed by blood sucking or applying traditional medicine (locally known as *40 KAMILI)*, or ash, mixed with water. Moreover, there were victims that were forced to vomit through oral administration of salt water or a mixture of charcoal and water. Other victims were given sheep’s oil to drink to give them strength before getting to the hospital. In most cases (>98%), initial first aid was given by individuals without any training in conventional medicine on snakebites and relevant first aid measures. In one of the cases, an old man (>60 years) drank a solution of *survidim*, a drug used for treating trypanosomiasis and babesiosis in cattle, sheep, and goats, after he was bitten and stayed home for a month before seeking hospital treatment. In other communities, ingestion of sugar and onion was also common practice as a first aid measure for snakebite victims. In 95% of cases the victims sought some form of further treatment at a hospital (43%) or through using traditional treatments (53%) made from a variety of locally available plants (Table 3). Eighty percent of victims who sought hospital treatment at the Meserani Snake Park Clinic received treatment with anti-venom (AV).

*Snake encounters*. The study communities are vulnerable to snakebites because of their way of life as agro-pastoralists and the high density of snake species both venomous and non-venomous in their surroundings. This makes it difficult to avoid snake encounters when herding cattle in the bush, especially during dry the season, when more snakes are around. Traditionally, the herders are barefoot (children) or are wearing open-toed sandals made from car tyres, providing no protection from bites. Snakes have also been reported to enter houses, presumably looking for food (rodents), and being startled by inhabitants moving in their sleep or when getting up at night. Snakes are often not seen due to lack of a light source.

Some community members interviewed knew how to recognise venomous snakes, but most did not. Those who did, relied on information from their elders when identifying dangerous snakes. During the interviews, villagers suggested training on snakes and snakebite treatment and management to help the victims once bitten.

#### Attitudes

*At risk communities*. In a bid to find ways to improve the availability of treatment to victims, participants were asked for their views on how the situation could be improved. The majority of the victims or their relatives considered equipping village dispensary and health care facilities with AV as the most promising intervention. Primary health centers were available in the villages; however, they did not have any AV in stock. Despite the habit of using traditional healers, all respondents mentioned that they were willing to shift from traditional medicine reliance if hospital treatments were easily available.

## Discussion

This study examined characteristics of snakebite victims in northern Tanzania and the health seeking behaviour of victims and their parents/guardians. It also looked at treatment challenges and the capacity of medical staff to treat snakebite, and collected suggestions for possible improvements. Overall, it became clear that the risk of snakebite differs significantly between age, gender, season and the activity carried out. Challenges when seeking treatment include recognizing venomous snakes, giving first aid and reaching a health care centre that has antivenom (AV) and trained personnel available. Interviewed health professionals reported lack of training and drugs for treatment as the main barriers for providing adequate care to snakebite victims.

Pastoralist communities in the Monduli and Simanjiro districts in northern Tanzania are vulnerable to snakebite, because of their way of life. Their simple homes are surrounded by dry bushland, representing perfect snake habitat, and, herding cattle, which involves walking vast distances every day, remains the main form of livelihood to over 65% of the people. Homesteads (Bomas) are often in remote areas, not connected to the electric power grid and far from tarmac roads and hospitals. Snakebite in these communities is common, but receiving appropriate medical treatment is challenging because of bad infrastructure, limited treatment capacity and a culture of relying on traditional treatment.

We obtained previously recorded snakebite case data from the Meserani Snake Park Clinic and from Monduli district Hospital (2007–2019). The privately run Meserani Snake Park Clinic in our study area was founded after the need for treatment was recognized [13]. It is a nurse-led clinic [14] and their database on snake bite cases from 2007 to 2019 shows a drastic increase in the number of victims throughout the years, with adults more often affected than children and males more than females. This gender and age distribution was confirmed by the results obtained from interviews with the victims from the communities. The underlying cause is most likely due to gender and age-separated activities in Masai societies [4], where men herd cattle and therefore walk long distances with open toed sandals through snake habitat. The results are in line with studies looking at risk factors for snakebite carried out in Ghana [15] and in Nigeria [16]. The similarity maybe greatly influenced by the culture of these communities, whereby males of these study populations spend much time in the fields herding and farming, while women perform other domestic activities. These results correspond to studies conducted in other parts of East Africa, for instance in Kenya, where more men were reported to have been bitten [9], too. In other societies, however, for instance in Cameroon, females were found to be mostly affected, which the researchers linked to the fact that it is mainly women working in the fields [17]. The same has been found in several studies in Asia [5].

Since records began, 1386 cases were reported by the Meserani Snake Bite Clinic until the end of 2019. However, we cannot conclude that this increase is only due to an increase in incidence, but also due to the growing reputation of the Meserani Snake Clinic and the lack of treatment offered elsewhere [13]. The records likely do not show the full picture because in sub-Saharan Africa, not all victims attend hospital, either because they do not seek medical treatment, die before hospital intervention is possible, or cannot afford hospital treatment as reported by [18]. Unfortunately, a low interest in the snakebite problem in Tanzania by the scientific community was found during literature review for this study and was also reported by the nurses at the clinic, emphasizing the neglect of this health issue.

The species composition of the snakes responsible for the bite stayed the same over the years and corresponds to the species identified previously in the area [13]. However, since 2016 the number of unidentified species increased.

Most of the victims interviewed in this study (53%), reported to have sought after-bite care from traditional healers, which are very respected in the area. Commonly applied treatment practices include the use of tourniquets, human urine, or cow’s milk for washing the bite site. Some victims were given sheep’s fat to drink when they were bitten, others were given salt water or water with charcoal to induce vomiting. A local traditional medicine sold by herbalists known as *40 KAMILI* is also frequently used. It is applied to the site of the bite after an incision is made to open the wound. A study in South Africa showed that communities also use traditional methods to treat snakebite [19]. Some of the practices reported the use of the victim’s urine to treat the bite. Also, there were times when traditional healers used various parts of cremated snakes; the burnt snake is ground into powder and ingested by licking it or drinking it with water. It was sometimes also applied directly onto the site of the bite.

In our study, snakebite envenomation is overrepresented among adults between 18 to 60 years (54%), which is the socially active group, followed by 5 to 10-year-olds (23%), thus affecting production activities and growth of these communities. A study done in Rwanda showed that the highest number of cases occurred among children (< 18 years) and young adults (18–30 years) what was attributed to the large population of children relative to adults in Rwanda [20]. Young adults were also affected in South America, Brazil [21]. The increased risk to economically productive age-groups, especially low-income rural workers, is most likely linked to their living environment and lack of preventative measures (shoes, torches, snake-proof housing) [22].

Snakebite incidences were significantly higher during the dry season (96%) compared to the rainy season (4%) in our study area. This could be explained by scarcity of water resources in which the few remaining permanent ponds become the interface for humans, cattle, and wildlife [13]. However, it should be noted that in many other parts of Africa research shows snakebite incidences were higher during the rainy season [17]. It seems to depend on the environmental conditions and the exposure of the victims due to the type of activities carried out. In our study area it was suggested that snakebite incidences are high during the dry season because this is the cooler time of the year and snakes, especially puff adders which are common in the area, come out to bask in the sun and are in search of water [23] This is also the period for harvesting crops and travel further with livestock across dry grazing areas, activities which increase the time spent working outside among agro-pastoralists, thus increasing snakebite incidences [4].

Unsurprisingly, being outdoors is a significant risk factor for snakebite among agro-pastoralists. Most of the victims (69%) were out in the bush and on their farms carrying out their daily activities such as herding their cattle when they were bitten.

Snake envenomation leads to severe short-term physical effects such as intense pain, vomiting, dizziness up to losing consciousness. However, long-term physical effects, were also reported by more than half of the victims in our study. The mortality rate was 9.6% and 45% of victims experienced long term pain either at the bite site or elsewhere in the body, which affected their ability to carry out daily duties. The same was reported in a review of clinical literature on the long-term effects of snake envenoming by [24]. Other long-term symptoms reported by the victims in our study were numbness especially during cold weather, permanent loss of sensation at the affected body part, mental disturbances, body swelling, hyperpigmentation, oozing from the bite site, severe headaches, dizziness, fever, sweating, tremor, nausea, and general body weakness. In many cases several of these symptoms were reported. Some of the victims died due to envenomation, for instance, a family from Loiborsoit village in Monduli district, lost two sons in a span of six months, both bitten as they were herding the family cattle in the field. Other adverse health effects may result from the application of traditional treatments [19]. Snakebite victims may also have long-term psychological implications such as anxiety, depression, and post-traumatic stress disorder (PSTD), which may result in social disadvantage and loss of employment due to disability [10]. These further underscores the burden of the mental, physical and socio-economic impact of snakebite on the victims and their communities.

In most cases, the snake species could not be identified by the villagers. About one third of people questioned, said they can identify snakes. However, other studies showed that often all snakes are regarded as venomous by these communities [4].

The study participants also described the challenges faced when seeking treatment. These challenges were poor transport services in terms of cost and bad roads especially during the rainy season. Lack of first aid kits for snakebite and AV in the village dispensaries forces victims to travel many kilometers to *Duka Bovu (Meserani Snake Park Clinic)* for treatment. This delays treatment of the victims, thus putting them at higher risk of serious health outcomes. Similar challenges have been reported in other countries like Kenya [25] and South America [26], thus increasing the severity of the disease and its effects in the communities.

Health centers in the villages lack both appropriate supportive treatment for snakebite, and AV, this is partly caused by unaffordability of AV and drugs. Village health centers also lack proper AV storage facilities [21, 27]. Not only the village dispensaries, but also government referral hospitals in these districts lack anti-venom and hence victims are only given first aid and referred to the clinic in Meserani. Additionally, medical staff we interviewed (69%) lacked knowledge and training on how to manage and treat snakebite. This was also found in Kenya [25] and in Cameroon [28] The only lifeline for snakebite victims in our study area is proper management and treatment at the Meserani Snake Park Clinic. However, accessibility of the Meserani Snake Park Clinic remains challenging for some of the communities due to poor infrastructure in their villages and lack and cost of reliable transport services.

The medical staff interviewed in this study emphasized the need for reduction in the incidence of snakebite by raising community awareness on the risks, and prevention by wearing appropriate footwear. These actions have been proposed in the WHO’s latest guidelines for the management of snakebite in Africa, recognizing that community education is the most effective preventive measure [29]. The staff also recommended improvements in the training of medical personnel and in the education of medical students through refresher courses on how to handle snakebite victims. This would enable correct administration of the available AV required by the victims. The medical staff also suggested educating the community to enable them to administer first aid and equipping the village health centres with first aid kits for easy and immediate access whenever needed. Transport to the nearest hospital can take hours, even days. It is therefore envisaged that by building capacity of village heath care providers many lives will be saved and effects of snakebite reduced. Standard protocols and tools for diagnosis and treatment of snakebite were suggested by the clinicians. This has been recommended by other scholars [3, 30] and in the recently updated WHO guidelines. In areas of high venomous snake species abundance and snakebite incidence where hospitals or clinics are poorly equipped or sometimes completely absent, the allocation of AV to health posts at the primary-care level should be considered. This should include integrated programs ensuring maintenance of proper storage facilities, appropriate training of health staff, and provision of AV and other drugs (including adrenaline, antihistamines and steroids) required for the management of envenoming. The preparation of national and regional snakebite treatment and management guidelines is of relevance for Tanzania, in order to ensure proper care for the victims.

During the study, the villagers were asked for suggestions on what could be done to help reduce the snakebite burden in their communities. Training on knowledge on snakes and treatment practices for snakebite was among the suggestions. Availability of first aid kits in the village dispensaries was also suggested. Additionally, they requested that AV is to be made available in the village dispensaries, to avoid having to travel long distances to get proper treatment. The study participants were all willing to shift from traditional medicine to modern medicine for effective treatment of snakebite. However, we cannot be certain that this result is representative of the general community, because of the study design which only interviewed victims and their relatives willing to take part in this scientific study. Other suggestions were introducing a power supply in the villages to be able to light their homes and streets at night time to avoid stepping on snakes and the improvement of infrastructure for reliable transport services across villages.

This study had some limitations due to limited time and resources. Hence, the exact prevalence of snakebite in the area could not be established, but for a first description of the situation, important information on incidence was collected. This study invited victims to be interviewed but to get a better picture on prevalence and the overall situation, a cross-sectional study should be used. Another limitation was the location of the villages were the snakebite victims lived. Some of the villages are very remote with bad roads, thus we could not reach some of the identified victims limiting the sample size. A bigger study with more resources would improve on this. We relied on reported facts with no way of checking the provided information. Future studies should try to implement a snake bite surveillance system which could form a foundation of reliable, scientifically proven information. Other limitations include recall bias and mistakes during translation and transcription of data and third-party reporting. Another issue was the lack of traditional healers in the study population. Hearing from traditional healers would have added vital information to the study. In conclusion, the vulnerabilities and challenges of the communities reporting snakebite are manifold. Snakebite is a neglected public health issue in northern Tanzania and more community led research is essential to find feasible short and long-term solutions. However, the first and foremost action to improve the situation is education for prevention of snakebite and the availability and storage of AV. Improvement to the currently available AVs in terms of reduced side effects or improved efficacy would be valuable. Antivenom against specific snake venoms are not currently available in Tanzania, and the available polyvalent AV may not be effective against bites from some snake species. Thus, production of AV against these snakes must be fast-tracked for immediate use. More research studies on the potential application of medicinal plant compounds of venom enzymes may result in improved and more effective treatment with reduced side effects for the snakebite victims [31]. This study emphasizes the extent of snakebite incidence and its effects, knowledge, practice and attitude towards snakebites, and also the challenges communities face in association with treatment. We have investigated the issues associated with prevention, treatment and management by consulting the victims of snakebite and the medical personnel involved. The findings of this study provide an evidence base for dialogue between researchers, the government, clinicians and the public in general. This paves the way to work together to achieve the crucial goals of the global snakebite initiative improved community education, improved education of medical personnel and improved research on effectiveness and safety of AV to the victims [32, 33].

## Supporting information

S1 Data (XLSX)

S1 File (DOCX)
