# Supplementary material for: Risks of snakebite and challenges to seeking and providing treatment for agro-pastoral communities in Tanzania
Source: PLoS One. 2023 Feb 10;18(2):e0280836. doi: 10.1371/journal.pone.0280836 (PMC9916632; doi:10.1371/journal.pone.0280836)
Supplement: S1 File — (DOCX) [file pone.0280836.s002.docx]

Supplementary Material for **“Risks of snakebite and challenges to seeking and providing treatment for agro-pastoral communities in Tanzania”**

**Snakebite cases by snake species:**

***SM 1:* Figure 2:** *Snakebites by snake species recorded from snakebite victims admitted at Meserani Snake Park Clinic from 2007 to 2019.*

***SM 2:* Figure 2:** *Distribution of snakebite incidences reported from the studied districts separated by season, with more incidences occurring more during the dry season.*

**SM 3: *Figure 4:*** *A graph showing the distribution of snakebite incidences by gender that were interviewed during the study from both districts.*

***SM 4:* Figure 5:** *Snakebite incidences by age group*

***SM 5: Questionnaire for victims or their guardians***

**General information**

Date and Time: ____/____/2021 ____: ____ a.m. /p.m.

Name: _____________________________________________________

Age: ___________________

Gender: ___________

Marital status: _________________________

Occupation: ___________________________

Education level___________________

Village: _____________________________________________________

**Risk factors**

1. Have any members of your household been bitten by a snake in the last 10 years?

a) Yes

b) No

c) Do not know

If yes, how often in the last ten (10) years? ____________________________________

2. Who in the household was bitten?

a) Self

b) Other__________________

3. What was/is the gender of victim?

a) Male

b) Female

4. What was the victim doing at the time? _________________________________

5. Do they usually do this?

a) Yes

b) No

6. How often do they do this?

a) Daily

b) Weekly

c) Monthly

d) Yearly

e) Once

7. Do you know which snakes are venomous?

a) Yes

b) No

What do they look like? ___________________________________

8. Site of bite

a) Hands and fingers

b) Feet

c) Leg

d) Other______________________________

9. Age of the household member at the time of bite age in years_______________

10. When were they bitten?

a) Last 1 year

b) Longer than 1 year ago but within the last ten years

11. What was the outcome?

a) Alive & fully recovered

b) Alive but not fully recovered

c) Died from snakebite

d) Died from causes other than snakebite

12. Can you tell me names of the different snakes that live here in this area?

a) Russell's viper

b) Cobra

c) Krait

d) Green Snake

e) Other (Specify)_____________________________________

13. During which time of the year do snakebites happen more often in this area?

a) Rain season

b) Dry season

c) Do not know

14. When did the incidence occur?

a) Morning

b) Noon

c) Evening

d) Night

15. In what places do most snake bites occur in this area?

a) Inside the house

b) Outside around (and close to) the house

c) Along a roadway/path/street

d) In a forest

e) In a field

f) Other (specify) _______________________________

g) Do not know

16. What were you doing during the incidence? _____________________

**Snakebite prevention, treatment and management practices**

17. What preventive measures do you use to prevent snakebite?

a) Wear proper shoes or boots and long trousers

b) Straight over rocks or logs rather than step on them

c) Use a light (torch, flashlight or lamp) when walking at night.

d) Avoid resting near the holes, nests and other hidden places

e) Have dog(s)

f) Other ___________________________________________

18. Do you teach kids how to avoid snake bite?

a) Yes

b) No

c) Sometimes

What do you tell them? _________________________________________

19. If someone from this village were bitten by a snake today, where would you first take them to get?

a) Treatment, including first aid?

b) Hospital

c) Traditional Healer

d) Would not take them anywhere

e) Other__________________________________

f) Do not know

UPP

- 1. 20. Who takes the victim?
  2. a) Anyone
  3. b) Family member

21. If the victim is a child who decides where to bring them?

a) Mother

b) Father

c) Other

22. Who brings the child for treatment?

a) Mother

b) Father

c) Other

23. For what reasons would you decide to take the snakebite victim to the place?

a) No cost to pay the health facility

b) Transport cost cheaper

c) Better quality of health care provided

d) Limited by the method of transport used

e) Shortest time to nearest health facility

f) They have antivenom

g) Other (specify)

24. What are the symptoms of snakebite?

a) Local bleeding and swelling

b) Severe pain at the site of the bite

c) Nausea and vomiting

d) Drowsiness and weakness

e) Dizziness

f) Other_______________

25. When handling dead snakes, people may suffer venom injection by an accidental scratch from the fang of a snake’s severed head?

a) True

b) False

26. Which of the following first-aid measures would you take if someone suffered a snakebite?

a) Immobilize the victim’s whole body, especially the wounded limb

b) Raise the site of the bite above the level of the person’s heart

c) Application of tight tourniquets around the upper part of the limb

d) Applying a pressure immobilization bandage

e) No response

27. What challenges do you encounter in accessing snakebite preventive or treatment measures? _________________________

28. What should change/be improved? ___________________________________________

29. Would you like to know more about snakes and bite treatment?

1. a) Yes
2. b) No

***SM 6: Questionnaire for health professionals***

**General information**

Date and Time: ____/____/2021 ____: ____ a.m. /p.m.

Name: __________________________________________

Age: ___________________

Gender: ____________________________

Profession: ___________________________

Education level________________________

Work station: __________________________________

**Knowledge on snakebites and envenomation**

1. For how long have you been working on snakebites? ______________ (in years)

2. Have you received any training on snakebite management?

a) Yes

b) No

c) If yes, who organized the training? ______________

3. What are the common snake species in the area?

(i)_______________________

(ii)_______________________

(iii)________________________

(iv)__________________________

4. Have you ever treated a snakebite patient?

a) Yes

b) No

If yes, can you distinguish between venomous and non-venomous snakebites?

1. a) Yes
2. b) No

5. Are you familiar with envenomation symptoms?

a) Yes

b) No

If yes, mention the common symptoms of envenomation

1. (i) _____________________________
2. (ii) _____________________________
3. (iii)_____________________________

**Snakebite prevention, treatment and management practices**

6. What preventive measures do you advise to prevent snakebite?

(i) ___________________________

(ii) ___________________________

(iii)___________________________

7. Do you provide trainings on snakebite treatment and management practices to the community?

a) Yes

b) No

c) If yes, how often? _________________________

8. What treatment measures do you give snakebite victims?

(i) ______________________

(ii) ______________________

(iii)______________________

(iv) ______________________

(v) ______________________

9. What are the factors affecting the availability and accessibility of AV?

(i) ______________________

(ii) ______________________

(iii)______________________

(iv) ______________________

(v) ______________________

10. What are the factors affecting the handling and storage of AV in the health facility?

(i) ______________________

(ii) ______________________

(iii) ______________________

(iv) ______________________

(v) ______________________

11. What challenges do you encounter when administering AV to snakebite victims?

(i) ______________________

(ii) ______________________

(iii)______________________

(iv) ______________________

(v) ______________________
